# Impact of respiratory syncytial virus disease on quality of life in adults aged ≥50 years: A qualitative patient experience cross‐sectional study

**DOI:** 10.1111/irv.12929

**Published:** 2022-01-03

**Authors:** Desmond Curran, Eliazar Sabater Cabrera, Benjamin Bracke, Kimberly Raymond, April Foster, Cindy Umanzor, Philibert Goulet, John H. Powers

**Affiliations:** ^1^ Value Evidence Vaccines GSK Wavre Belgium; ^2^ Scientific Consulting QualityMetric Johnston Rhode Island USA; ^3^ Department of Clinical Medicine George Washington University School of Medicine & Health Sciences Washington District of Columbia USA; ^4^ Present address: AstraZeneca Cambridge United Kingdom; ^5^ Present address: Incyte Corporation Morges Switzerland

**Keywords:** InFLUenza Patient Reported Outcome (FLU‐PRO), qualitative research, quality of life, respiratory syncytial virus

## Abstract

**Background:**

Information about the impact of respiratory syncytial virus (RSV) on quality of life in older adults is limited. This study characterized the patient experience of RSV illness in USA older adults and assessed the content validity of the InFLUenza Patient Reported Outcome (FLU‐PRO) in this population.

**Methods:**

This qualitative, non‐interventional, cross‐sectional study included hybrid concept elicitation and cognitive debriefing interviews with 30 individuals (age ≥50 years) with polymerase chain reaction‐confirmed RSV diagnosed within 6 months of screening. Targeted literature review was first conducted to inform the development of interview materials. Webcam or telephone interviews were conducted by qualitative researchers using a semistructured interview guide. Interview transcripts were coded and analyzed using Excel and NVivo software.

**Results:**

All participants reported impacts on daily activities, social activities, and relationships during RSV disease. Physical functioning was impaired in 25 (83%) participants, and 18 (60%) reported not engaging in leisure activities/hobbies. All nine participants who were working reported major impacts on work. Most (n = 28; 93%) described emotional impacts. A majority (n = 19; 63%) reported symptoms lasting beyond the acute disease stage from a week to >1 month. Symptom concepts reported generally matched FLU‐PRO items and domains. Cognitive debriefing indicated that FLU‐PRO was easy to understand and captured participants' experiences of RSV illness.

**Conclusions:**

This study indicates that RSV disease in adults aged ≥50 years in the USA has substantial impacts on daily life and that the concepts included in FLU‐PRO are appropriate and fit for purpose as a measure of RSV symptoms in this population.

**Abbreviations**
COPD: chronic obstructive pulmonary disease
FDA: Food and Drug Administration
FLU‐PRO: InFLUenza Patient Reported Outcome
PRO: patient‐reported outcome
QoL: quality of life
RSV: respiratory syncytial virus

## 1 INTRODUCTION

Human respiratory syncytial virus (RSV) is a major pathogen in pediatric populations, and reinfection may occur throughout life. Given similar host response, common symptoms of RSV infection are similar to those of other respiratory viruses, including cough, blocked/runny nose, sore throat, and trouble breathing.^1^ Research in older adults (specifically, aged ≥65 years) indicates RSV as an important pathogen in winter respiratory illness.^2^ In the United States of America (USA), annual RSV incidence is 1–7% in adults aged ≥50 years.^3^, ^4^

The risk of RSV infection requiring medical attention^4^ or hospitalization/emergency department attendance^5^ increases with age, and a majority of individuals aged 50–64 years, hospitalized with RSV, report ≤1 chronic comorbidity.^6^ Groups at high risk of severe RSV illness include older adults (especially aged ≥65 years), adults with chronic heart or lung disease, and adults with weakened immune systems.^7^ RSV infection requiring hospitalization in older adults may result in prolonged functional decline. A recent study of 39 patients aged ≥60 years, hospitalized with RSV infection in the USA, and with functional assessment data at 2 months of follow‐up showed that 23% of older adults required a higher care level at discharge, and activities of daily living scores were decreased at 2 months compared with baseline in 36%.^8^ Frail older adults are especially vulnerable to functional decline following infection.^9^ However, although RSV illness is recognized as an important disease in older adults, there is limited qualitative evidence exploring older adult patients' experiences with RSV illness and its impact on quality of life (QoL).

Exploring the symptoms and QoL impact of RSV illness in older adults needs a valid patient‐reported outcome (PRO) measure that can capture patients' experiences of RSV. The InFLUenza Patient Reported Outcome (FLU‐PRO; Leidos Biomedical Research, USA) is a PRO measure developed to assess symptoms of viral respiratory illness in adults.^10^ The measure contains 32 items in six domains, which evaluate the severity, in the past 24 h of symptoms, in the nose (four items), throat (three items), eyes (three items), chest/respiratory (seven items), gastrointestinal (four items), and body/systemic (11 items), and has been shown to produce scores that are well defined, reliable, valid, and responsive to change in influenza‐positive and influenza‐negative adults.^11^, ^12^ Clinical symptom concepts are similar across respiratory viral illnesses in older adults, indicating that the FLU‐PRO could be suitable for use in RSV. The reliability and validity of FLU‐PRO as an outcome measure has been demonstrated in a Phase II clinical trial of an experimental RSV vaccine in adults aged ≥60 years.^13^ This indicates that the FLU‐PRO measurement properties have been validated in an older adult RSV population, although qualitative content validation has been lacking until the present study. Concept elicitation and cognitive debriefing are recommended as important steps toward establishing content validity in any PRO measure by the USA Food and Drug Administration (FDA)^14^ and the leading professional society for health economics and outcomes research, the International Society for Pharmacoeconomics.^15^

The objectives of the present study were twofold: first, to characterize the patient experience and QoL impacts during and after an episode of RSV illness in USA older adults (aged ≥50 years) and identify concepts of importance to measure in this population, to inform the development of a conceptual model of RSV illness, including both proximal and distal impacts. Proximal impacts refer to health effects resulting directly from RSV symptoms, for example, difficulty in breathing having an impact on physical functioning. Distal impacts refer to those beyond the direct results of RSV symptoms, such as impacts on emotional or social functioning. The second objective is to assess the content validity of the FLU‐PRO as a tool for capturing the patient experience (symptom presence, severity, and frequency) of RSV illness in older adults.

## 2 METHODS

### 2.1 Study design

**FIGURE 1:**
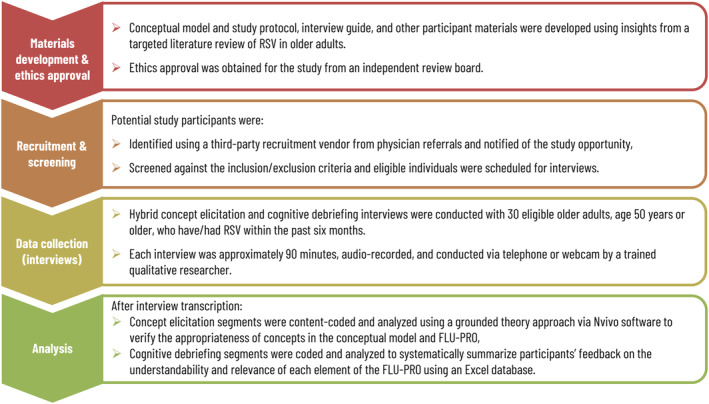
Study design. FLU‐PRO, InFLUenza Patient Reported Outcome; RSV, respiratory syncytial virus

This qualitative, cross‐sectional, non‐interventional, observational study included hybrid concept elicitation and cognitive debriefing interviews with 30 individuals (age ≥50 years) who had RSV within 6 months of screening. A targeted literature review was first conducted to inform the development of the conceptual model, the study protocol, and supporting materials for the qualitative interviews that were subsequently submitted to an independent review board. Figure 1 shows an overview of the study design.

### 2.2 Literature review

PubMed was searched for articles published between September 2009 and September 2019 that focused on patient‐reported concepts of RSV burden, impacts on QoL, risk factors, signs, symptoms, treatments, and comorbid or chronic conditions associated with RSV (Appendix S1). In addition, relevant sources from the reference lists of selected manuscripts were reviewed, and unstructured searches using Google Scholar and RSV support/advocacy group websites were used to fill in any gaps.^1^ Only English‐language publications were included.

### 2.3 Sample size and saturation analysis

The sample size was estimated with the intent to reach saturation,^14^, ^17^ when further interviews no longer introduce new concepts or themes. Previous research has suggested that 97% of symptom concepts emerge by the 20th interview.^18^ As this study included sample quotas related to age, ranging from 50 to over 80 years, the sample size was set at 30 participants to maximize the likelihood of capturing at least 97% of key concepts within an older adult population. Saturation analysis was conducted using a process examining concepts and themes across sets of consecutive interviews.^15^, ^17^, ^19^ Transcripts were coded in six sets of five transcripts each. An initial coding structure was determined after coding the first set and modified to account for new concepts that emerged while coding each subsequent set.

### 2.4 Recruitment and screening

Potential participants were screened by telephone, and those who were interested were asked to provide a physician‐confirmed polymerase chain reaction diagnosis of RSV, received within the past 6 months, to be eligible. Participants were also required to be aged ≥50 years; live in the USA; be able to read, write, and fully understand the English language; and be willing and able to participate in an interview for 90 min.

### 2.5 Study procedures

All participants read and signed an informed consent form prior to their scheduled interview. Interviews were conducted between January 8, 2020, and March 20, 2020, by experienced and trained qualitative researchers using a semistructured interview guide. The guide included a list of instructions for the interviewer to ensure that each interview was conducted consistently. All interviews were conducted via telephone or webcam and audio recorded. Following the concept elicitation segment of the interview, which explored participants' descriptions of their experiences of RSV, a cognitive debriefing exercise was conducted where participants completed the FLU‐PRO using the think‐aloud method.^20^ Participants then answered a set of structured queries, to gather patient feedback on all elements of the FLU‐PRO, including instructions, items, response options, and recall period.

Audio files of interviews were transcribed verbatim. All interview transcripts were content coded by two trained qualitative analysts and confirmed by the principal investigator. Three transcripts (10%) were double coded to ensure reliability between coders. Discrepancies between coders were reviewed, discussed, and resolved by the research team in scheduled consensus meetings.

### 2.6 Concept elicitation

Concept elicitation data were coded and analyzed using constant comparison^21^, ^22^ in accordance with grounded theory analysis methods.^17^, ^23^ Concepts emerging from participants, that is, rather than imposing an a priori theory, were identified. All transcripts were reviewed using NVivo qualitative research software (QSR International Pty Ltd., Version 12, 2019). The concepts and themes in participants' descriptions of their experiences of RSV informed the conceptual model and were subsequently used to map participants' experiences to the symptoms included in the FLU‐PRO.

### 2.7 Cognitive debriefing

Cognitive debriefing data, from the same set of patients, were coded and analyzed using a Microsoft Excel database to systematically summarize participants' feedback on the understandability and relevance of the instrument. Codes were assigned to each patient‐reported issue according to its likely impact on comprehension and validity of data and whether the issue was reported spontaneously by participants.

## 3 RESULTS

### 3.1 Demographic and clinical characteristics

A total of 30 interviews were conducted, and the demographic and clinical characteristics of the respondents are summarized in Table 1.

**TABLE 1:** Demographic and clinical characteristics of study participants

Demographics and clinical characteristics	Number (N = 30)	%
Current age (years)		
50–64	15	53
65–79	12	37
80+	3	10
Time since RSV episode		
1 month	10	33
2 months	10	33
3 months	5	17
6 months	5	17
Sex		
Male	15	50
Female	15	50
Race/ethnicity		
White	13	43
Other (American Indian or Alaska Native, Black/African American, Hispanic or Latino, Native Hawaiian/Other Pacific Islander)	17	57
Geographical location		
USA—North	4	13
USA—South	6	20
USA—East	6	20
USA—West	14	47
Treatment setting		
Outpatient (physician office, urgent care)	22	73
Hospital/emergency room	8	27
Diagnosis of comorbid condition^a^		
Asthma	6	20
Chronic obstructive pulmonary disease	9	30
Congestive heart failure	5	17
Other (hypertension, emphysema, irritable bowel syndrome)	2	7
None	12	40

Abbreviations: RSV, respiratory syncytial virus; USA, United States of America.

^a^ Participants reported multiple comorbid conditions.

The sample included 15 individuals 50–64 years of age, 12 individuals 65–79 years of age, and 3 individuals ≥80 years of age (Table 1). Most participants (n = 18; 60%) reported at least one comorbid condition such as asthma, chronic obstructive pulmonary disease (COPD), congestive heart failure, hypertension, emphysema, and irritable bowel syndrome representing the real‐world population with RSV disease. Most participants (n = 22; 73%) had been treated in the outpatient setting. Eight (27%) had been treated in the hospital/emergency room, including four adults aged ≥65 years who had been hospitalized.

### 3.2 Saturation analysis

Table S1 summarizes the number of codes identified in each set of transcripts. Of the 65 codes identified in total, 58 (89%) were identified in the first set and a further 5 (8%) in the second and third sets. Few new concepts of importance emerged in the last sets, suggesting that 30 interviews were sufficient to reach saturation.

### 3.3 Signs and symptoms

Table 2 presents the RSV symptoms reported by participants during the interview, spontaneously or when probed. Symptoms generally matched those listed in the FLU‐PRO. Gastrointestinal symptoms such as diarrhea and vomiting were reported by 12 (40%) and 7 (23%) participants, respectively. These symptoms were concentrated in participants aged ≥65 years; 10 (83%) reports of diarrhea and 5 (71%) reports of vomiting were in this age group.

**TABLE 2:** Reported symptoms mapped to FLU‐PRO items

FLU‐PRO symptom	Total reported^a^ N = 30	Total reported^a^ %	Spontaneously reported N = 30	Spontaneously reported %
Congested or stuffy nose	30	100	12	40
Weak or tired	30	100	13	43
Coughing	30	100	26	87
Chest congestion	29	97	3	10
Trouble breathing	29	97	19	63
Body aches or pains	29	97	16	53
Headache	28	93	13	43
Lack of appetite	28	93	5	17
Coughed up mucus or phlegm	28	93	10	33
Head congestion	27	90	1	3
Runny or dripping nose	26	87	16	53
Chills or shivering	26	87	3	10
Felt cold	25	83	3	10
Sinus pressure	24	80	2	7
Dry or hacking cough	24	80	8	27
Wet or loose cough	24	80	5	17
Sleeping more than usual	24	80	4	13
Sore or painful throat	23	77	11	37
Sweating	23	77	2	7
Sneezing	23	77	3	10
Difficulty swallowing	22	73	1	3
Scratchy or itchy throat	22	73	3	10
Felt hot	22	73	0	0
Teary or watery eyes	21	70	1	3
Chest tightness	21	70	0	0
Felt dizzy	21	70	1	3
Eyes sensitive to light	18	60	0	0
Felt nauseous	14	47	4	13
Sore or painful eyes	12	40	1	3
Diarrhea	12	40	5	17
Stomachache	8	27	0	0
Vomit	7	23	5	17

Abbreviations: FLU‐PRO, InFLUenza Patient Reported Outcome; RSV, respiratory syncytial virus.

^a^ Total includes number of participants who identified experiencing symptom during RSV infection spontaneously or when probed.

The symptoms reported as most bothersome were coughing (n = 12; 40%), trouble breathing (n = 11; 37%), fever or feverish (n = 8; 27%), and body aches or pains (n = 7; 23%). There were 52 signs/symptoms reported in total, 20 of which were not described by participants using the same language as FLU‐PRO items, such as fever or feverish, fatigue or lack of energy, wheezing, shortness of breath, chest pain, ear pain or pressure, and hoarseness. However, as confirmed by patients during cognitive debriefing, many of these additional symptoms were captured by existing items in FLU‐PRO. For example, all participants reporting wheezing (n = 13) and shortness of breath (n = 12) reported that “trouble breathing” in the FLU‐PRO captured these symptoms during the qualitative interviews. Specifically, 29 patients (97%) reported “trouble breathing”; among them, 19 (63%) were spontaneously reported (Table 2). This would suggest that “trouble breathing” captures the concept of “shortness of breath” and that “trouble breathing” was used more commonly by the participants themselves to describe this symptom.

Similarly, 27 (90%) participants reported fever or feeling feverish, a multidimensional concept^24^ that is included in the FLU‐PRO but divided up among multiple concepts such as feeling cold or hot, shivering or chills, headaches, or sweating, which the patients used to describe their experience.

### 3.4 Impacts on QoL

**FIGURE 2:**
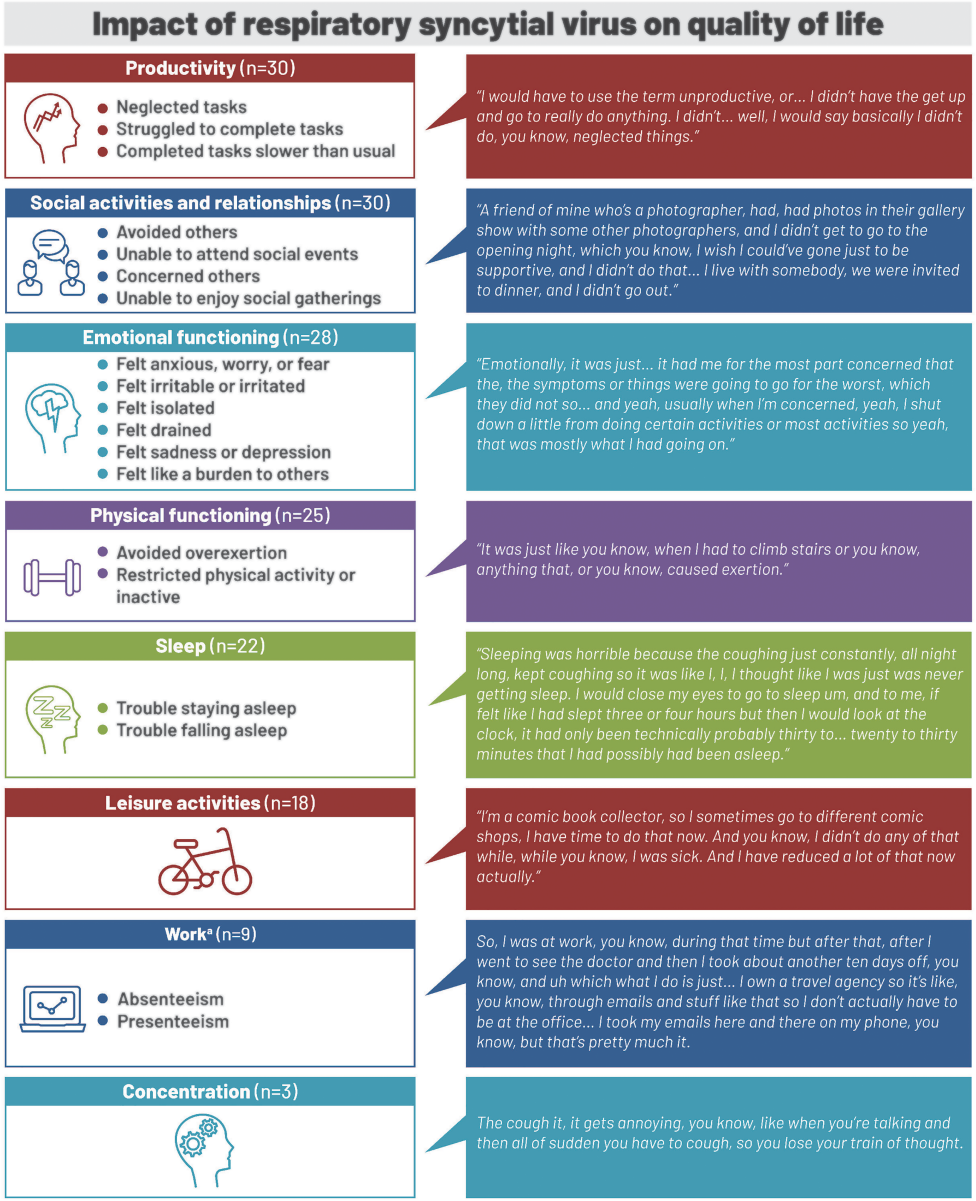
Reported impact of respiratory syncytial virus illness on quality of life. ^a^Nine participants were working at the time of the respiratory syncytial virus infection

All 30 participants reported impacts of RSV illness on productivity. Most (n = 24; 80%) could not leave the house during active RSV illness, and in the home, participants reported neglecting chores, or limited ability or taking longer to complete day‐to‐day tasks such as showering or preparing meals. All 30 participants reported impacts on social activities and relationships; 26 (87%) described avoiding others, and 25 (83%) canceled social plans. Figure 2 summarizes reported impacts of RSV illness on QoL, with example quotations to illustrate the participants' experiences.

Emotional impacts were reported by 28 (93%) participants, for example:

Emotionally, it was just … it had me for the most part concerned that the, the symptoms or things were going to go for the worst ….

Some participants feared that they might have pneumonia or other serious illness. Symptoms such as trouble breathing and gastrointestinal symptoms such as vomiting were particularly concerning for participants. Some participants aged >65 years or with exacerbations of coexisting conditions due to RSV infection described fears of dying or returning to hospital, for example:

It's RSV, first I did not know what it is. I mean, I, I ask him [treating physician] if it's dangerous, and my kids, I can die for this because I'm uh 69.

Other emotional impacts included feeling irritable/irritated/frustrated (n = 9; 30%), emotionally drained (n = 8; 27%), or depressed (n = 4; 13%) because of the severity and duration of symptoms, and four (13%) felt isolated due to being housebound and avoiding others.

Physical functioning was impaired in 25 (83%) participants, for example:

I ride my bike for exercise, since I'm retired, I, I didn't ride my bike that day, so, when I had the … when I had the illness or sickness, I, I didn't ride the bike, I, I didn't feel up to it.

Eighteen (60%) reported decreased vitality that resulted in not engaging in leisure activities or hobbies. All nine participants who were working reported major impacts on work, for example:

So, I was at work, you know, during that time but after that, after I went to see the doctor and then I took about another ten days off ….

Time off work ranged from 3 days to 3 weeks, typically on the advice of a healthcare provider. Those who remained at work or returned before symptoms had fully resolved reported reduced productivity and efficiency at work due to lack of energy. Sleep disruptions at night were reported by 22 (73%) participants, for example:

Sleeping was horrible because the coughing just constantly, all night long, kept coughing ….

A majority of participants (n = 19; 63%) reported symptoms, such as cough, throat irritation, and tiredness, that lingered beyond the acute disease stage from a week to over a month and reported that it was more difficult to recover from RSV than previous respiratory illnesses they had experienced. Half of the participants (n = 15) described current impacts, at the time of the interview, on physical functioning, leisure activities, productivity, relationships or social activities, and emotional functioning.

### 3.5 Treatment experiences

Most participants managed the symptoms of their RSV illness with over‐the‐counter (n = 25; 83%) and/or prescribed medications (n = 20; 67%), mainly for cough (n = 21; 70%), fever (n = 18; 60%), and trouble breathing (n = 16; 53%). Of the eight (27%) participants who required treatment at a healthcare facility, five required treatments to improve breathing due to symptoms related to trouble breathing, shortness of breath, and wheezing.

### 3.6 Cognitive debriefing: Participants' feedback on FLU‐PRO

All participants reported that the FLU‐PRO instructions were easy to understand, and the 24‐h recall period would be easy to remember. Most (n = 27; 90%) indicated that the length was appropriate. The majority of participants (n = 22; 73%) reported they would be able to complete the questionnaire during different stages of RSV illness, whereas eight (27%) reported that it may be difficult to complete during the worst phase of their RSV illness.

**FIGURE 3:**
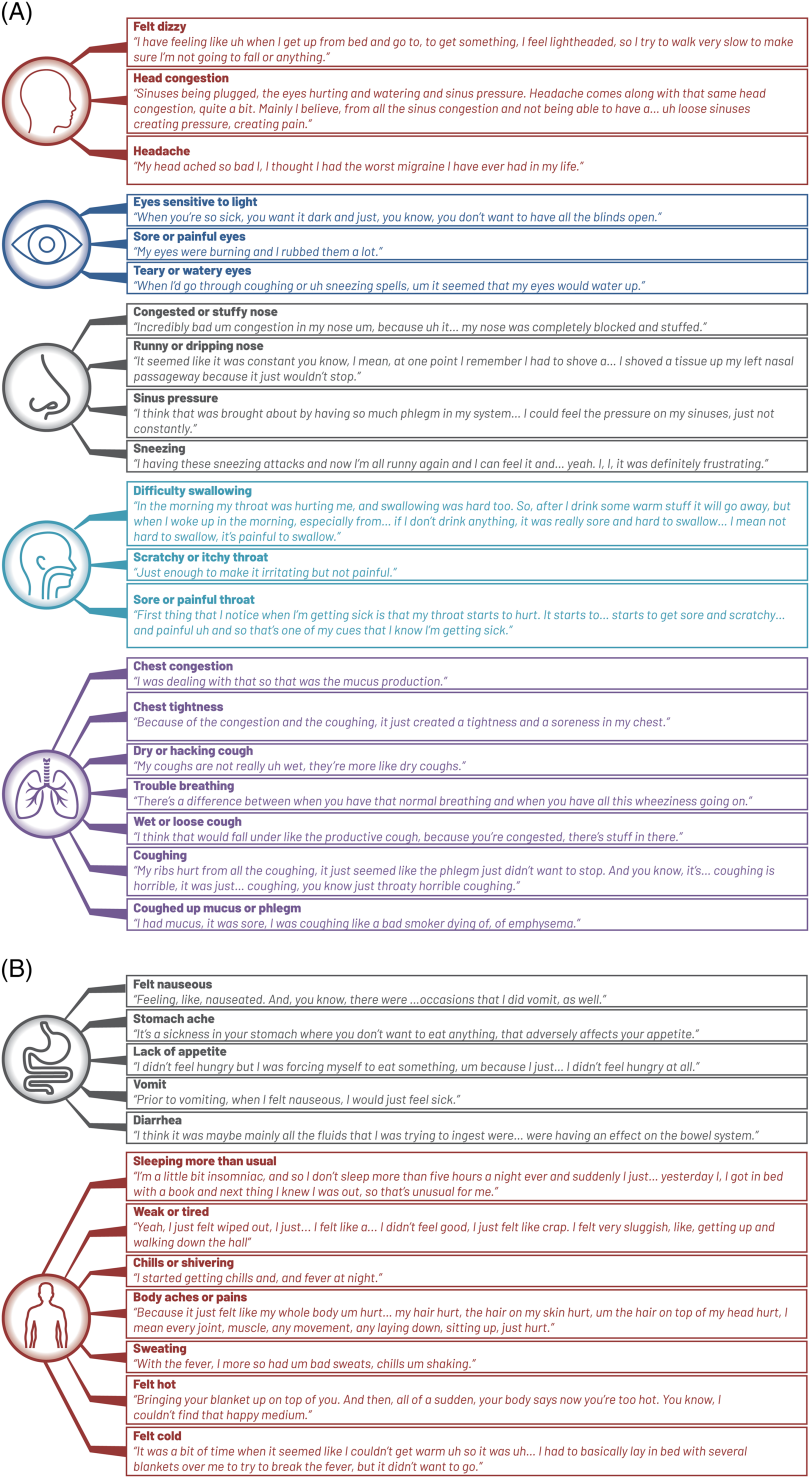
Participant quotations illustrating symptom experiences mapped to InFLUenza Patient Reported Outcome items: (A) respiratory‐specific body system and (B) gastrointestinal and general symptoms

Most reported that the items accurately reflected their RSV symptoms. Of the 32 symptoms in the FLU‐PRO instrument, 29 were reported as relevant by at least half the participants (Table S2). Gastrointestinal symptoms such as stomachache (47%), vomiting (37%), and diarrhea (47%) were less commonly reported. Eight participants (27%) reported difficulty with answering “stomachache,” because they considered their abdominal muscles being sore or tight from coughing, having an empty stomach from lack of appetite, or medication side effects. Two participants reported that the FLU‐PRO did not miss any symptoms, but it would be helpful to include additional questions to capture their whole experience including impacts on daily life, treatment experiences, illness phases encountered, and their environment during their RSV illness. These participants enjoyed sharing their experience with the interviewer and wanted a way to capture this full experience as part of the instrument. Figure 3 provides examples of participant quotations to illustrate their experience of the FLU‐PRO items.

Item content analysis indicated that the FLU‐PRO was generally easy to understand and answer. For example:

It's short, self‐explanatory, it's not too complicated, they are phrases or … sentences used on it, it's mostly on the basic English side. So that makes it understandable, easy to read, easy to comprehend and easy, easy to answer.

The FLU‐PRO includes three sets of response options evaluating concept intensity and frequency:

- not at all, a little bit, somewhat, quite a bit, and very much;
- never, rarely, sometimes, often, and always; and
- 0 times, 1 time, 2 times, 3 times, and 4 or more times.

All participants found the second and third sets easy to understand, and all except one found the first set easy to understand.

Nineteen of the 29 participants who were asked (66%) reported that the FLU‐PRO comprehensively captured their experiences with no missing symptoms. Eight different participants reported missing symptoms, but these symptoms varied across the interviews. Three participants mentioned preferring the inclusion of one specific question regarding fever rather than answering the fever symptoms listed in the FLU‐PRO like “felt hot,” “felt cold,” and “chills or shivering”; however, participants were accurately able to describe their fever using these existing items in the FLU‐PRO. Eight additional signs and symptoms were reported, each by a single participant, and included wheezing, general malaise, dry skin, constipation, itchy nose, fatigue, lack of energy, and chest pain. Although the participants described the sound of their breathing as “wheezing,” which is a sign not a symptom, what they felt was the symptom of “trouble breathing.” As wheeze is a medical term used to describe the presence of adventitious sound, and which may also be confused with other sounds such as crackles and rhonchi, it is more appropriate that this sign is assessed by a trained medical practitioner, rather than self‐reported in a PRO. Even in a medical setting, there can be disagreement on the classification of these sounds.^25^ Some of these signs and symptoms, such as general malaise and lack of energy, are already included in the FLU‐PRO using different language.

### 3.7 Final conceptual model

**FIGURE 4:**
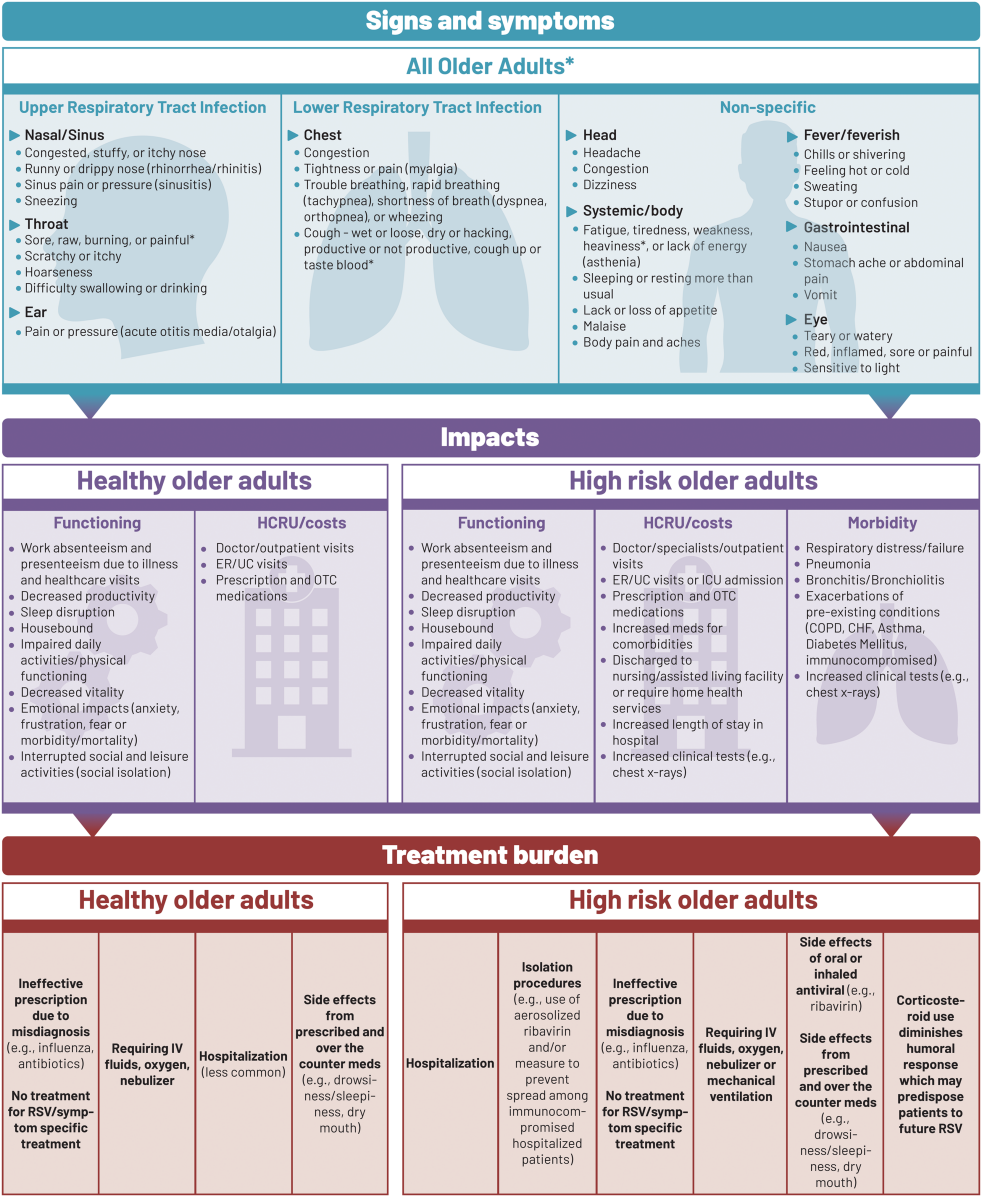
Conceptual model: Signs, symptoms, impacts, and treatment burden. *High‐risk individuals experience more severe symptoms and complications. CHF, congestive heart failure; COPD, chronic obstructive pulmonary disease; ER, emergency room; HCRU, healthcare resource use; ICU, intensive care unit; IV, intravenous; meds, medications; OTC, over‐the‐counter; RSV, respiratory syncytial virus; UC, urgent care

The draft conceptual model developed from the literature review was modified and refined using findings from the qualitative interviews. The final conceptual model summarizes the causes, transmission, and risk factors (Figure S1) and the symptoms, impacts, and treatment burden (Figure 4) of RSV illness on healthy and high‐risk older adults. This provides a visual representation of important concepts in patients' experience of RSV disease and the relationship between the concepts, including symptoms, treatment burden, proximal QoL impacts such as physical functioning, and distal QoL impacts such as effects on emotions and social relationships.

## 4 DISCUSSION

This qualitative study used hybrid concept elicitation and cognitive debriefing interviews to explore patients' experiences of RSV disease in individuals aged ≥50 years in the USA and to relate the experiences reported by participants to the items in the FLU‐PRO. The study also evaluated the content validity of the FLU‐PRO as a tool for capturing the patient experience of RSV illness in older adults. To our knowledge, this is the first qualitative validation of the FLU‐PRO in this population. Participants reported a wide range of impacts of RSV disease on QoL. Proximal impacts, directly related to the symptoms of RSV disease, included impacts on physical functioning or the impact of coughing on sleep. More distal impacts included effects on emotional functioning, such as anxiety about the potential seriousness of the illness, and time away from work. The information presented here captures a broad spectrum of patients' experiences with RSV disease, extending beyond symptoms to wider impacts on patients' daily lives. This broader understanding of patients' experiences may help healthcare professionals to better support patients with RSV disease.

Symptoms captured in the concept elicitation part of the interview generally matched the items in the FLU‐PRO, and the cognitive debriefing confirmed that the participants found the FLU‐PRO easy to understand and answer and that it comprehensively captured their experiences of RSV illness. Gastrointestinal symptoms such as stomachache, vomiting, and diarrhea were less commonly reported and thus may be less commonly associated with RSV but are still relevant to the disease. As a result, some participants defined these symptoms differently to fit in with their RSV experience. Diarrhea and vomiting were concentrated in the group aged ≥65 years. In older adults, especially in those with comorbidities, and/or frail individuals, infectious diseases may exacerbate issues associated with polypharmacy, leading to more adverse events and gastrointestinal symptomatology.^26^ As such, it is valuable and clinically meaningful to capture these symptoms (and/or the reduction of these symptoms). The FLU‐PRO may be used in conjunction with generic questionnaires such as the Short Form 36 Health Survey (SF‐36v2). Consequently, the FLU‐PRO is not developed to capture all domains that are more distal impacts of the disease, for example, physical functioning, and/or QoL concepts such as effects on emotions and social relationships, which may be captured using other instruments.

Guidance for industry issued by the FDA indicates how PRO measures should be developed and used to measure the impact of an intervention on aspects of patients' health status, ranging from purely symptomatic to complex concepts such as QoL.^14^ This guidance recommends that the exact words used in the instrument to represent the concepts should reflect patient input. The research presented here suggests that the signs and symptoms described by patients with RSV in the concept elicitation interviews generally matched the items in the FLU‐PRO and that language used in the FLU‐PRO such as “trouble breathing” was commonly used by patients to describe their experience. The evidence from the present study supports FLU‐PRO as an appropriate tool that is fit for the purpose of measuring RSV symptoms experienced by people aged ≥50 years in the USA.

One of the strengths of the present study was the qualitative interview approach, which allows an enhanced understanding of the patients' overall experience of illness extending beyond symptoms. Further strengths included the broad age range and gender balance of the sample and the inclusion of participants treated in a range of healthcare settings. Another strength was the inclusion of individuals with comorbid conditions, such as asthma or COPD, because a majority of patients with RSV have comorbidities.^6^ Therefore it was important to include patients with comorbidities in the study to represent the real‐world experiences of such patients. Most participants were recruited within 2 months of diagnosis to help alleviate recall bias. The inclusion of participants up to 6 months after diagnosis was intended to capture participants' reports of information on longer term impacts of disease.

Despite the strengths of this research, limitations should also be acknowledged. The concept elicitation interviews served to identify important concepts related to the experience of RSV, in particular impacts of RSV, rather than to measure frequency of impacts. However, the goal of qualitative research is not to establish frequency of elicited concepts. As most interviews were conducted by telephone, interviewers were not able to respond to visual cues such as body language or gestures. However, this limitation was mitigated as interviewers were trained to listen carefully for non‐verbal cues such as pauses or sounds that could indicate confusion and to probe for clarification if necessary.

In conclusion, the results of this qualitative study indicate that RSV disease in adults aged ≥50 years in the USA resulted in significant impacts on patients' daily lives, including impacts on productivity inside and outside the home, social activities and relationships, emotional functioning, physical functioning, sleep, and leisure activities. The study also provides evidence that symptoms reported by the participants match items captured in the FLU‐PRO questionnaire and that the FLU‐PRO is appropriate and fit for purpose as a measure of RSV symptoms in adults aged ≥50 years. This qualitative study provides valuable information about patients' experience of RSV disease and the use of FLU‐PRO in this population.

## AUTHOR CONTRIBUTIONS

**Desmond Curran:** Conceptualization; formal analysis; investigation; methodology; supervision; validation; visualization. **Eliazar Sabater Cabrera:** Conceptualization; formal analysis; methodology; validation; visualization. **Benjamin Bracke:** Conceptualization; methodology; validation; visualization. **Kimberly Raymond:** Conceptualization; data curation; formal analysis; investigation; methodology; supervision; validation; visualization. **April Foster:** Conceptualization; data curation; formal analysis; investigation; methodology; validation; visualization. **Cindy Umanzor:** Conceptualization; data curation; formal analysis; investigation; methodology; validation; visualization. **Philibert Goulet:** Conceptualization; methodology; validation; visualization. **John H. Powers III:** Conceptualization; methodology; software; validation; visualization.

### PEER REVIEW

The peer review history for this article is available at https://publons.com/publon/10.1111/irv.12929.

## Supporting information


**Figure S1:** Conceptual model: causes, transmission and risk factors

**Table S1:** Saturation tracking

**Table S2:** FLU‐PRO items reported as relevant and most relevant

**Appendix S1:** Search strategy

## Data Availability

GSK makes available the anonymized individual participant data and associated documents from interventional clinical studies, which evaluate medicines, upon approval of proposals submitted to www.clinicalstudydatarequest.com. To request access to patient‐level data and documents for this study, please submit an enquiry via www.clinicalstudydatarequest.com. Information on GSK's data sharing commitments and requesting access to anonymized individual participant data and associated documents can be found at www.clinicalstudydatarequest.com.

## ENDNOTE

1 A report from the National Foundation for Infectious Diseases was added to the literature review using this approach.^16^
