# Supplementary material for: Impact of respiratory syncytial virus disease on quality of life in adults aged ≥50 years: A qualitative patient experience cross‐sectional study
Source: Influenza Other Respir Viruses. 2022 Jan 3;16(3):462–73. doi: 10.1111/irv.12929 (PMC8983922; doi:10.1111/irv.12929)
Supplement: Supplementary file 1 — Figure S1: Conceptual model: causes, transmission and risk factors [file IRV-16-462-s003.docx]

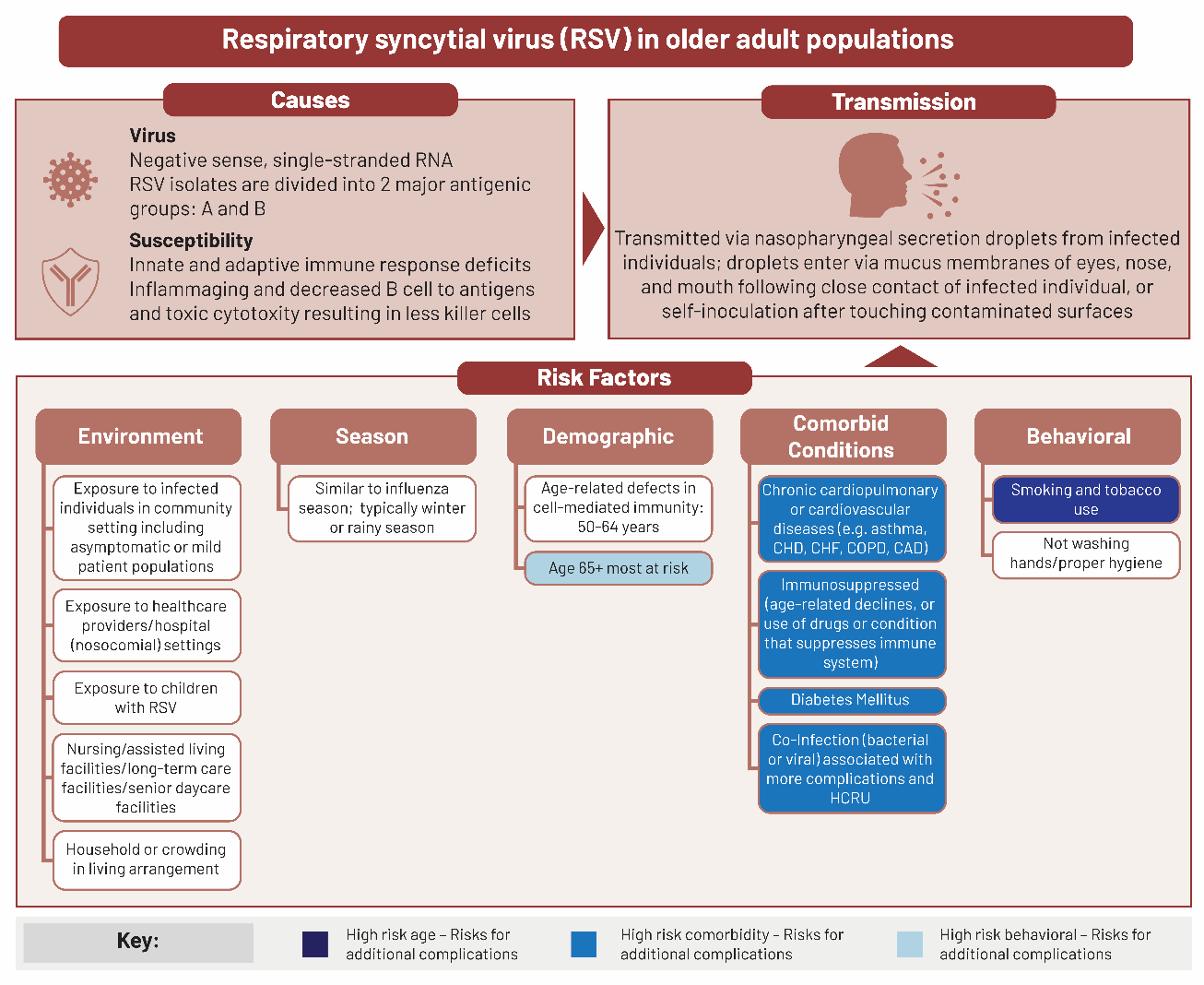
**Figure S1**: Conceptual model: causes, transmission and risk factors

CAD, coronary artery disease; CHD, coronary heart disease; CHF, congestive heart failure; COPD, chronic obstructive pulmonary disease; HCRU, healthcare resource use; RNA, ribonucleic acid; RSV, respiratory syncytial virus
