# Supplementary material for: Impact of respiratory syncytial virus disease on quality of life in adults aged ≥50 years: A qualitative patient experience cross‐sectional study
Source: Influenza Other Respir Viruses. 2022 Jan 3;16(3):462–73. doi: 10.1111/irv.12929 (PMC8983922; doi:10.1111/irv.12929)
Supplement: Supplementary file 2 — Table S1: Saturation tracking [file IRV-16-462-s004.docx]

**Table S1**: Saturation tracking

| **Major theme** | **Code set 1 (Interviews 1-5)** | **Code set 2 (Interviews 6-10)** | **Code set 3 (Interviews 11-15)** | **Code set 4 (Interviews 16-20)** | **Code set 5 (Interviews 21-25)** | **Code set 6 (Interviews 26-30)** |
| --- | --- | --- | --- | --- | --- | --- |
| Signs or symptoms | 46 | 1 | 3 | 1 | 0 | 1^†^ |
| Impacts | 9 | 0 | 1 | 0 | 0 | 0 |
| Treatment experiences | 3 | 0 | 0 | 0 | 0 | 0 |
| Total | 58 | 1 | 4 | 1 | 0 | 1 |

^†^The symptom identified was confusion, which was reported by two participants who were over the age of 80. They associated this symptom with a fever and being put out of their usual routine while being hospitalized.
