# Supplementary material for: Impact of respiratory syncytial virus disease on quality of life in adults aged ≥50 years: A qualitative patient experience cross‐sectional study
Source: Influenza Other Respir Viruses. 2022 Jan 3;16(3):462–73. doi: 10.1111/irv.12929 (PMC8983922; doi:10.1111/irv.12929)
Supplement: Supplementary file 3 — Table S2: FLU‐PRO items reported as relevant and most relevant [file IRV-16-462-s002.docx]

**Table S2**: FLU-PRO items reported as relevant and most relevant

| **FLU-PRO symptom** | **# of participants reported relevant**^†^ | **Reported relevant %** | **# of participants reported most relevant**^†^ | **Reported most relevant %** |
| --- | --- | --- | --- | --- |
| Runny or dripping nose | 28 | 93 | 16 | 53 |
| Congested or stuffy nose | 28 | 93 | 10 | 33 |
| Sinus pressure | 26 | 87 | 8 | 27 |
| Scratchy or itchy throat | 24 | 80 | 6 | 20 |
| Sore or painful throat | 24 | 80 | 5 | 17 |
| Difficulty swallowing | 23 | 77 | 3 | 10 |
| Teary or watery eyes | 23 | 77 | 3 | 10 |
| Sore or painful eyes | 18 | 60 | 2 | 7 |
| Eyes sensitive to light | 19 | 63 | 2 | 7 |
| Trouble breathing | 29 | 97 | 18 | 60 |
| Chest congestion | 29 | 97 | 12 | 40 |
| Chest tightness | 25 | 83 | 7 | 23 |
| Dry or hacking cough | 26 | 87 | 13 | 43 |
| Wet or loose cough | 27 | 90 | 14 | 47 |
| Felt nauseous (feeling like you wanted to throw-up) | 18 | 60 | 1 | 3 |
| Stomach-ache | 14 | 47 | 1 | 3 |
| Felt dizzy | 26 | 87 | 2 | 7 |
| Head congestion | 26 | 87 | 3 | 10 |
| Headache | 30 | 100 | 8 | 27 |
| Lack of appetite | 30 | 100 | 7 | 23 |
| Sleeping more than usual | 27 | 90 | 7 | 23 |
| Body aches or pains | 29 | 97 | 8 | 27 |
| Weak or tired | 30 | 100 | 14 | 47 |
| Chills or shivering | 28 | 93 | 8 | 27 |
| Felt cold | 26 | 87 | 7 | 23 |
| Felt hot | 26 | 87 | 7 | 23 |
| Sweating | 25 | 83 | 6 | 20 |
| Sneezing | 27 | 90 | 9 | 30 |
| Coughing | 30 | 100 | 22 | 73 |
| Coughed up mucus or phlegm | 30 | 100 | 11 | 37 |
| Vomit | 11 | 37 | 1 | 3 |
| Diarrhea | 14 | 47 | 2 | 7 |

^†^Participants could report multiple symptoms.

FLU-PRO, InFLUenza Patient Reported Outcome
