# Supplementary material for: Impact of respiratory syncytial virus disease on quality of life in adults aged ≥50 years: A qualitative patient experience cross‐sectional study
Source: Influenza Other Respir Viruses. 2022 Jan 3;16(3):462–73. doi: 10.1111/irv.12929 (PMC8983922; doi:10.1111/irv.12929)
Supplement: Supplementary file 4 — Appendix S1: Search strategy [file IRV-16-462-s001.docx]

## Appendix S1. Search strategy

## Information sources

We searched PubMed for articles describing respiratory syncytial virus (RSV) and focused on concepts reported by the patient such as the burden of RSV, impacts of RSV, and quality of life of patients with RSV. In addition, we reviewed the literature for risk factors, signs, symptoms, and treatments as well as comorbid or chronic conditions associated with RSV. Articles identified encompass qualitative and quantitative findings. The search was limited to articles published between September 2009 and September 2019 ("2009/09"[Date - Publication] : "2019/09"[Date - Publication.)

Articles from the PubMed literature search were reviewed, as well as additional, relevant sources gleaned from the reference lists of selected manuscripts. Unstructured searches using Google Scholar and RSV support or advocacy group websites were leveraged to fill in any gaps regarding signs, symptoms, treatments, and risk factors; the only one in which publications were found to add to the literature review was a report from the National Foundation for Infectious Diseases.^16^

## Search strings for PubMed search

The following four structured searches resulted in a preliminary 223 records (excluding duplicates).

**Search 1: Patient reported concepts of RSV experience**

The following preliminary search resulted in 89 records.

1. “respiratory syncytial virus” OR “RSV”
2. “older adult” OR senior OR aged OR “elderly adult” OR elderly NOT (child* OR infant)
3. qualitative OR interview OR “focus group*” OR elicitation OR questionnaire OR survey OR subjective OR “patient reported” OR outcomes
4. "2009/09"[Date - Publication] : "2019/09"[Date - Publication]
5. (#1 AND #2 AND #3 AND #4)

**Search 2: Review articles of signs and symptoms of RSV**

The following preliminary search resulted in 4 records.

1. “respiratory syncytial virus” OR “RSV”
2. “older adult” OR senior OR aged OR “elderly adult” OR elderly NOT (child* OR infant)
3. signs OR symptoms
4. "2009/09"[Date - Publication] : "2019/09"[Date - Publication]
5. (#1 AND #2 AND #3 AND #4)
6. Apply Filter: Review

**Search 3: Burden of RSV and quality of life**

The following preliminary search resulted in 83 records.

1. “respiratory syncytial virus” OR “RSV”
2. “older adult” OR senior OR aged OR “elderly adult” OR elderly NOT (child* OR infant)
3. "burden" OR "disease burden" OR "burden of disease" OR "patient burden" OR "patient impact" OR "burden of illness" OR "quality of life" OR "impact" OR “health-related quality of life”
4. "2009/09"[Date - Publication] : "2019/09"[Date - Publication]
5. (#1 AND #2 AND #3 AND #4)

**Search 4: Comorbid conditions associated with RSV**

The following preliminary search resulted in 143 records.

1. “respiratory syncytial virus” OR “RSV”
2. “older adult” OR senior OR aged OR “elderly adult” OR elderly NOT (child* OR infant)
3. comorbid* OR asthma OR "chronic obstructive pulmonary disease" OR “COPD” OR pneumonia OR "heart failure" OR “cardiovascular disease” OR “CVD” OR bronchitis
4. "2009/09"[Date - Publication] : "2019/09"[Date - Publication]
5. (#1 AND #2 AND #3 AND #4)

## Screening of abstracts

Abstracts for each record were reviewed in order to select articles for full-text review. Criteria for exclusion of a record from full-text review:

- Article is not available in English
- Article describes a protocol for a future study
- Study sample consists of animals rather than humans
- Content of the abstract indicates that the article is irrelevant to the current objective (e.g., abstracts that mention RSV, but do not provide any substantive information that would contribute to the conceptual model; abstracts that do not focus on the experience of the condition in older adults)

## Full-text review of articles

The full-text of all articles meeting the above-listed selection criteria were reviewed in their entirety. In all, 64 full-text articles were reviewed. Upon full-text review, 10 articles were excluded from the final review because the content was methodological in nature or otherwise did not provide useful content for this study. Reviewers looked specifically to identify findings to inform further development of a conceptual model of RSV, as well as information that could support the development of the patient interview guides.

To ensure consistency and quality during the review process, 3 reviewers were trained on how to review and document each article in alignment with the study objectives. All reviewers assessed the same 3 articles and the principal investigator examined uniformity in the data extraction across reviewers. During the subsequent review of articles, if the results from an article were unclear, a second reviewer also assessed the publication to help determine the material to be extracted. As part of this quality control process, the principal investigator randomly chose 10% of articles to be re-reviewed to confirm the consistency and quality of the data extractions.

Additionally, the targeted literature review focused on articles that document the development and validation of the InFLUenza Patient Reported Outcome (FLU-PRO). The results of this review were used to gain a broader understanding of the impact RSV has in adults 50 years of age or older, utilize concept findings to develop a draft version of the conceptual model of RSV in older adults and support the development of the qualitative study interview guide and materials.
